# Multi-trait polygenic risk scores improve genomic prediction of atrial fibrillation across diverse ancestries

**DOI:** 10.21203/rs.3.rs-7713077/v1

**Published:** 2025-10-03

**Authors:** Sean Jurgens, Poeya Haydarlou, Daria Kramarenko, Marre Corver, Nobuyuki Enzan, Dominic Zimmerman, Patrick Ellinor, Connie Bezzina

**Affiliations:** Amsterdam UMC; Amsterdam UMC; Amsterdam UMC; Amsterdam UMC; Broad Institute of MIT and Harvard; Amsterdam UMC; Broad institute; Heart Center, Department of Clinical and Experimental Cardiology, Amsterdam UMC, location AMC

## Abstract

Polygenic scores (PGSs) can improve atrial fibrillation (AF) risk prediction, both alone and alongside clinical scores, offering potential for guiding targeted screening. However, their limited accuracy and cross-ancestry transferability remain major barriers to clinical translation. Here, we explored several multi-PGS approaches to generate ancestry-optimized PGSs for AF, and tested these in independent samples from the All of Us Research Program. Our ancestry-specific multi-trait approach, which leverages correlated traits, in particular outperformed the current gold-standard PGS among Asian (OR/SD = 1.76 [1.56–1.99]; AUROC = 0.637; AUPRC = 0.055), Admixed American (1.45 [1.38–1.53]; 0.595; 0.054) and African ancestry groups (1.39 [1.32–1.45]; 0.573; 0.064). Although predictive accuracy remained highest among Europeans (1.89 [1.85–1.93]; 0.646; 0.157) - in whom our PGS explained ~ 50% of SNP-heritability - our multi-trait approach yielded relatively larger gains in non-European populations. Improved risk stratification was also observed at PGS extremes, identifying a substantial proportion of European individuals with risk comparable to rare *TTN* variants (e.g., 5.8% with > 4-fold odds). Overall, our ancestry-tailored multi-trait PGSs advance equitable AF risk prediction and provide a foundation for implementation.

## Introduction

Polygenic scores (PGSs) have gained considerable interest as tools to predict an individual’s genetic propensity to diverse phenotypes and diseases^1–3^. Generated from large genome-wide association studies (GWAS), PGSs aim to condense multiple common risk alleles into a singular quantitative score^4^. As GWAS sample sizes have grown and PGS prediction has improved, PGSs have garnered substantial interest for potential clinical implementation. Indeed, as of late 2025, Mass General Brigham (MGB) hospital has started offering a PGS testing service, allowing for genetic risk estimation across eight common diseases, among which atrial fibrillation (AF)^5,6^.

AF is the most common sustained cardiac arrhythmia, affecting over 30 million people worldwide^7^. AF is associated with over three-fold risk of both ischemic stroke and heart failure (HF)^8,9^, making it a major contributor to global cardiovascular morbidity and mortality. Large GWAS have successfully established hundreds of common risk loci for AF^10^, and have estimated that common genetic variants explain over 22% of variance in AF susceptibility^11^. Subsequently, PGSs have been developed that aim to estimate genetic risk of AF. In a landmark paper, Roselli et al. built an AF PGS from a GWAS of around 180,000 AF cases, which showed notable prediction of incident AF when used in conjunction with age and sex (C-index ≈ 0.8), and arguably serves as the current gold standard^12^. As opposed to clinical models (such as the CHARGE-AF score^13,14^), a PGS may indicate risk long before traditional clinical risk factors emerge, highlighting their potential for early prediction and prevention^15^.

In spite of the current movement towards clinical implementation, however, PGSs face several major barriers that limit their clinical and research utility. For instance, current AF PGSs show only modest predictive accuracy and lack clearly-defined clinical use-cases, limiting their potential as prediction tools for actionability in the clinic. Perhaps more importantly, current PGSs transfer poorly across ancestries, with reduced accuracy in non-European populations, which may lead to worsening of existing inequities in healthcare. The moderate transferability in part stems from a persistent lack of diversity in GWAS training datasets and a predominant focus on individuals of European descent^1^. This pattern is evident in existing PGSs for AF reported in the PGS catalog, as most are trained on European data only and are rarely validated across diverse ancestries.

Here, taking AF as exemplary trait, we sought to assess and address these challenges through three key strategies: First, we enhanced ancestral diversity of our training data by integrating GWAS summary statistics from the AFGen Consortium^12^ with the Million Veteran Program (MVP)^16^, the latter being distinguished by its broad ancestral representation comprising approximately 29% non-European individuals^17^. Second, we applied SBayesRC, a novel Bayesian framework that produces dense genome-wide PGSs that leverage functional genomic annotations to enhance prediction accuracy, particularly across ancestries^18^. Third, we investigated two complementary strategies for polygenic score integration: A multi-ancestry approach combining PGSs derived from different ancestral backgrounds, and a multi-trait approach integrating scores from AF and multiple correlated traits, similar to the GPSmult and PRSmix + methods, which improved coronary artery disease (CAD) prediction^2,19^ across various ancestry groups.

Using training data from 269,746 AF cases and nearly 10 million individuals, and leveraging GWAS data from correlated traits, we developed ancestry-optimized multi-PGSs and tested these in the diverse All of Us Research Program (AoU). Our goal was to assess and improve genomic prediction of AF across ancestries, thereby providing a more equitable and interpretable foundation for clinical translation.

## Results

### Creating multi-ancestry polygenic scores for AF

To generate novel PGSs for AF, we first meta-analyzed large-scale GWAS data from the AFGen consortium (N = 181,446 cases) with GWAS data from the MVP (N = 88,300 cases), creating large, ancestrally-diverse GWAS datasets for AF. Building on this, we applied the SBayesRC algorithm to the ancestry-specific GWAS summary statistics (EUR, AFR, AMR, EAS), resulting in EURmeta, AFRmeta, AMRmeta, and EASmeta scores; we also used SBayesRC to create an all-ancestry PGS (ALLmeta), using the pan-ancestry meta-analysis as training data (Fig. S1, Methods).

PGS tuning and validation, unless otherwise specified, was performed in the All of Us Research Program (Table S4), an ancestrally-diverse cohort from the United States. Interestingly, during PGS validation, the ancestry-specific scores did not consistently outperform the gold-standard, all-ancestry trained PGS from Roselli et al. This was reflected by ΔOR/SD values among EUR (+ 0.13), AFR (−0.04), and AMR (−0.26) validation cohorts (Fig. S2; Table S1). Therefore, we used the multi-framework from SBayesRC to combine each ancestry-specific score (e.g., EURmeta or AFRmeta) with the all-ancestry PGS (ALLmeta), using weights derived from the joint predictive performance in an independent dataset restricted to a single ancestry, i.e., an ancestry-specific tuning set (Fig. S1). The resulting multi-ancestry - or “Mult-a” - scores outperformed the Roselli PGS across EUR, AMR and AFR ancestry in the validation set (Fig. S2). Specifically, the OR per SD increase of the PGS improved from 1.59 to 1.82 in EUR, from 1.21 to 1.30 in AFR, and from 1.34 to 1.39 in AMR. Interestingly, we found that the Mult-a scores showed nearly identical predictive accuracy relative to the ALLmeta score alone, showing similar prediction within EUR (ΔOR/SD: 0.00; ΔAUROC: +0.002), AFR (−0.02; +0.001) and AMR (0.00; 0.000) ancestry validation samples (Fig. S2; Table S1). Consequently, we continued model development using the simpler all-ancestry PGS (the ALLmeta score).

### Developing multi-trait polygenic scores for AF

**Figure 1:**
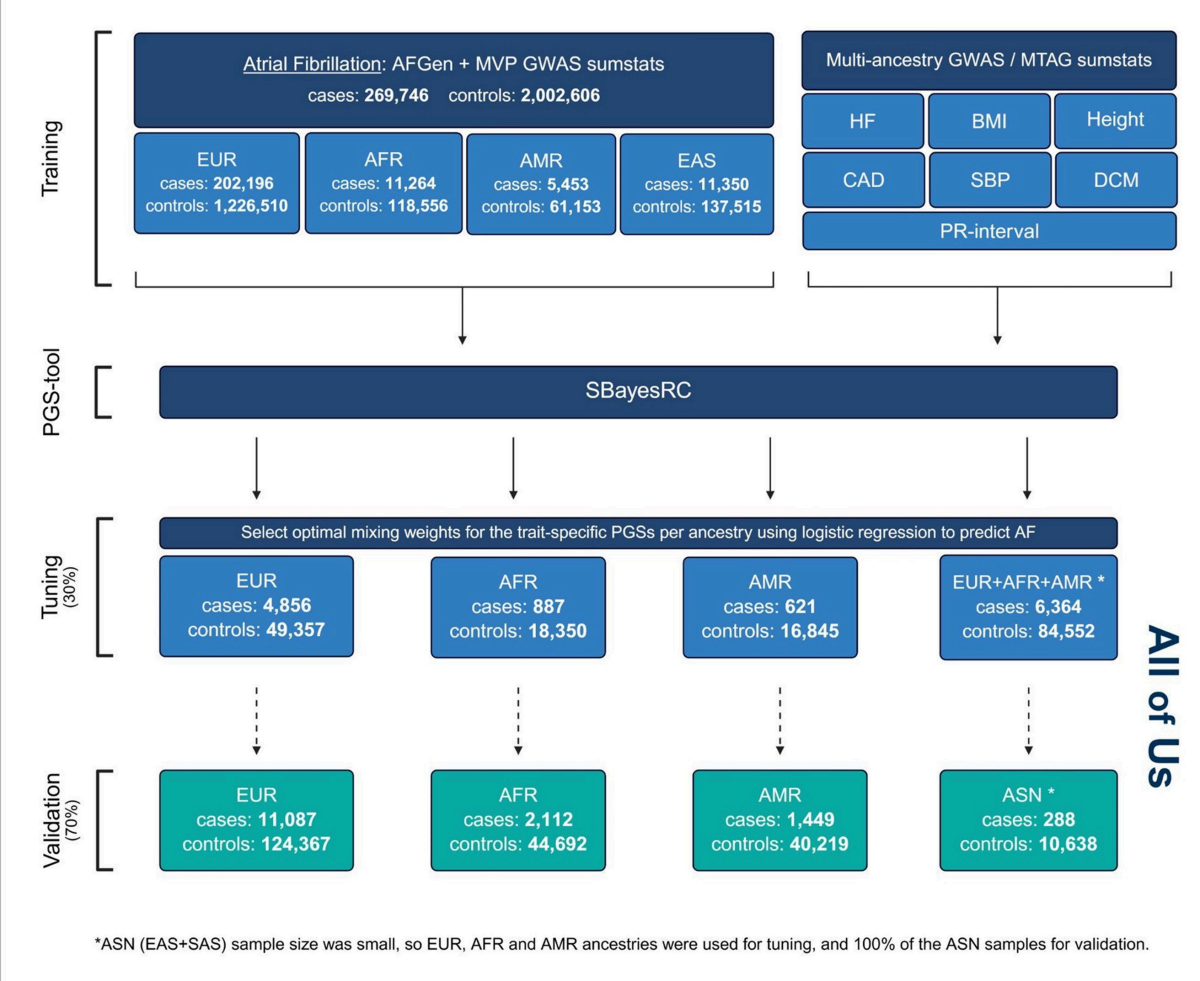
Mult-t polygenic scores development, an overview. This flowchart illustrates the development of the Mult-t polygenic scores across four key stages: Training, PGS tool, Tuning (30%), and Validation (70%), with the latter two performed in the All of Us dataset. The scores were derived from i) all-ancestry GWAS summary statistics for AF from the AFGen and MVP cohorts across four ancestral groups (EUR, AFR, AMR, and EAS; top left), and ii) all-ancestry training data from seven traits correlated with AF (top right). SBayesRC was used to generate polygenic score files for each trait. These were combined in four different ways using unadjusted logistic regression models to predict AF in each ancestry-specific tuning set, and weights were given based on that. The resulting ancestry-tuned multi-trait PGSs were evaluated in the corresponding ancestry-specific testing sets using logistic regression adjusted for the first 20 PCs, age and sex. Ancestries: EUR, European; AFR, African; AMR, Admixed American; EAS, East Asian; SAS, South Asian; ASN, Asian (EAS+SAS). Traits: HF, heart failure; BMI, body mass index; Height; CAD, coronary artery disease; SBP, systolic blood pressure; DCM, dilated cardiomyopathy. Created with BioRender.com.

In the next stage, we integrated the ALLmeta score with all-ancestry trained PGSs for seven traits correlated with AF, within a custom multi-trait approach (Fig. S3, Methods), similarly applying performance-based weights (or mixing weights) from a tuning set. We evaluated two tuning strategies for constructing the multi-trait - or “Mult-t” - scores: one using ancestry-specific tuning sets and another using the all-ancestry tuning set (Fig. S4, Methods). In the All of Us dataset, the ancestry-tuned, or ancestry-optimized, Mult-t scores consistently outperformed the all-tuned versions across the EUR (ΔOR/SD = 0.01) and AMR (0.03) validation cohorts, with the strongest improvement observed in the AFR group (0.07) (Fig. S5; Table S2). Based on these results, we selected the Mult-t-EUR, Mult-t-AFR, and Mult-t-AMR scores as the final models for the respective ancestries. For the Mult-t-ASN score, a unique tuning strategy was applied to ensure a sufficiently large validation set. We used a multi-ancestry tuning set excluding ASN individuals, enabling evaluation in a 100% ASN validation cohort. An overview of the final PGS construction, including case/control counts for the training, tuning, and validation datasets, is provided in a flowchart (Fig. 1). Additional details on the training data, including data for the correlated traits not shown in the figure, are available in Table S12.

### Enhanced prediction of atrial fibrillation using multi-trait polygenic scores

**Figure 2:**
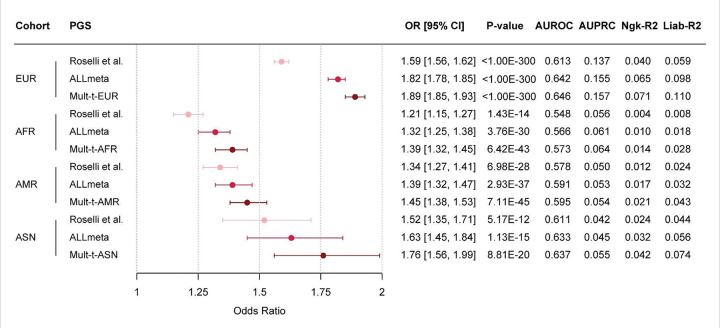
Comparison of the Roselli et al., ALLmeta, and Mult-t polygenic scores. The left panel displays a forest plot of the OR/SD increase in PGS for AF, with 95% CIs on the x-axis. The y-axis lists the ancestry-specific validation cohorts, ordered by decreasing cohort size, each paired with its respective PGSs. The right panel summarizes key performance metrics from the validation set. All estimates are based on logistic regression models adjusted for the first 20 PCs, age, and sex, using case/control counts from the 70% validation dataset of All of Us (Table S4). Due to limited sample size, the full 100% validation set was used for ASN ancestry. Model performance details are provided (Table S3).

We assessed performance of the final multi-trait PGSs (Mult-t) compared to the ALLmeta and Roselli et al. PGSs in the All of Us validation set (Fig. 2). Across all ancestry groups, the ALLmeta score outperformed the previously published Roselli PGS. The Mult-t scores demonstrated further improvements over the ALLmeta score, with notably the largest relative gains observed in the ASN (ΔOR/SD = 0.13; ΔAUROC = 0.004, ΔAUPRC = 0.010) and AFR (0.07; 0.007; 0.003) validation cohorts, followed by EUR (0.07; 0.004; 0.002) and AMR (0.06; 0.004; 0.001) (Table S3). These findings underscore the added value of incorporating correlated traits into polygenic scores, particularly for non-EUR ancestries.

As expected, multi-trait PGS performance was highest in the European validation set (OR/SD = 1.89 [1.85–1.93]; AUROC = 0.646 [0.641–0.652]; AUPRC = 0.157), followed by the ASN (1.76 [1.56–1.99]; 0.637 [0.603–0.672]; 0.055), AMR (1.45 [1.38–1.53]; 0.595 [0.580–0.610]; 0.054), and AFR (1.39 [1.32–1.45]; 0.573 [0.560–0.585]; 0.064) validation sets. The largest relative gains over the Roselli score were observed in Europeans (ΔOR/SD = 0.30; ΔAUROC = 0.033; ΔAUPRC = 0.020), followed by gains in ASN (0.24; 0.026; 0.013), AFR (0.18; 0.025; 0.008), and AMR (0.11; 0.017; 0.004) populations (Fig. 2; Table S3).

In addition, Mult-t scores achieved higher Nagelkerke’s R^2^ and liability-scale R^2^ than the Roselli score across validation sets, reflecting greater explanatory power among EUR (ΔNgk-R^2^ = 0.031; ΔLiab-R^2^ = 0.051), ASN (0.018; 0.030), AFR (0.010; 0.020), and AMR (0.009; 0.019) ancestries (Fig. 2; Table S3). Taken together, these results demonstrate that leveraging all-ancestry training data, paired with a multi-trait framework, improves AF risk prediction across ancestries compared to the existing gold standard. While absolute performance remains highest in Europeans, the multi-trait approach offers particularly meaningful improvements in non-EUR populations.

Finally, MGB will be implementing a PGS built using the PRSmix + approach^2^, which also uses a multi-trait framework. We therefore also assessed the currently available PRSmix + PGS for AF, making sure to exclude samples from AoU that were potentially used in model development (Fig. S6). We found that the PRSmix + PGS marginally outperformed the Roselli et al. score across ancestries, although notably our Mult-t PGSs clearly outperformed the available PRSmix + score (Fig. S6; Table S5).

### Decomposition of the Mult-t scores by trait

**Figure 3:**
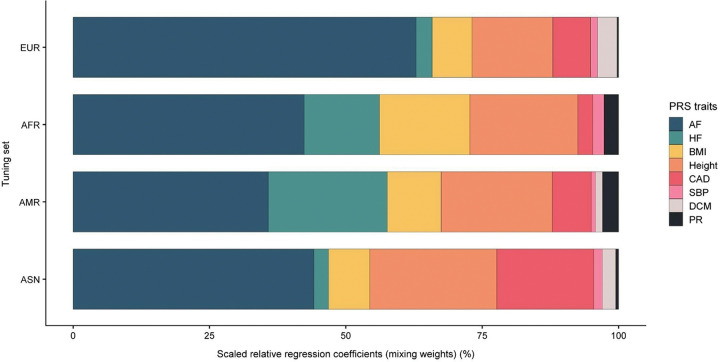
Scaled relative regression coefficients of the Mult-t PGSs across ancestry-specific tuning sets. The plot illustrates the relative contribution of each of the eight traits (legend) to the final Mult-t scores across ancestry-specific tuning sets (y-axis). The x-axis represents the mixing weights, scaled regression coefficients expressed as percentages and normalized to 100% to reflect the trait composition of the Mult-t PGSs. Mixing weights were derived from unadjusted logistic regression models based on each trait’s predictive accuracy for AF in the corresponding tuning set. Additional details are available (Table S6). Unscaled coefficients are provided in the supplements (Fig. S7).

To better understand the predictive basis of the Mult-t PGSs, we examined the components and relative contributions (Fig. 3). Each score is composed of eight individual PGSs, derived from all-ancestry GWAS summary statistics for AF and seven related traits. These component scores were weighted based on their predictive performance for AF in an ancestry-specific tuning set.

In each tuning set, the AF PGS contributed the most to the Mult-t score (35.8–62.9%) (Table S6). Height was the second-largest contributor in most ancestry groups (14.8–23.3%), except in AMR, where heart failure (HF) ranked second. The third-largest contributor varied across body mass index (BMI) (7.3–16.6%), HF (2.7–21.8%), and coronary artery disease (CAD) (2.7–17.8%). AF had the highest relative contribution in the EUR tuning set (62.9%) compared to the other ancestry groups, while BMI, HF, and CAD had the largest relative contribution in AFR (16.6%), AMR (21.8%) and ASN (17.8%) ancestries, compared to the other ancestry groups, respectively. Systolic blood pressure (SBP), dilated cardiomyopathy (DCM), and PR interval contributed minimally, in general (≤ 3.6%). Overall, the heterogeneity in trait contributions highlights that the correlated traits capture ancestry-relevant signals that are not fully reflected by the AF-derived PGS alone.

We also evaluated the trait-specific PGSs individually in the ancestry-specific validation sets of All of Us, to better understand each trait’s contribution to the final score. Results are shown in Figures S8–S11. AF PGS consistently showed the strongest predictive performance across all ancestries (OR/SD = 1.32–1.82), particularly in EUR (1.82) (Table S8). HF PGS ranked second (1.24–1.50) and BMI PGS third (1.17–1.35) in all ancestry groups. Height PGS performed better than CAD PGS and SBP PGS in EUR and ASN, whereas in AFR and AMR, CAD PGS and SBP PGS outperformed height (Figs S8-S11). DCM PGS and PR interval PGS had minimal predictive value across all ancestries (≤ 1.06).

### Validation of multi-trait PGSs in non-European samples of the UK Biobank

**Figure 4:**
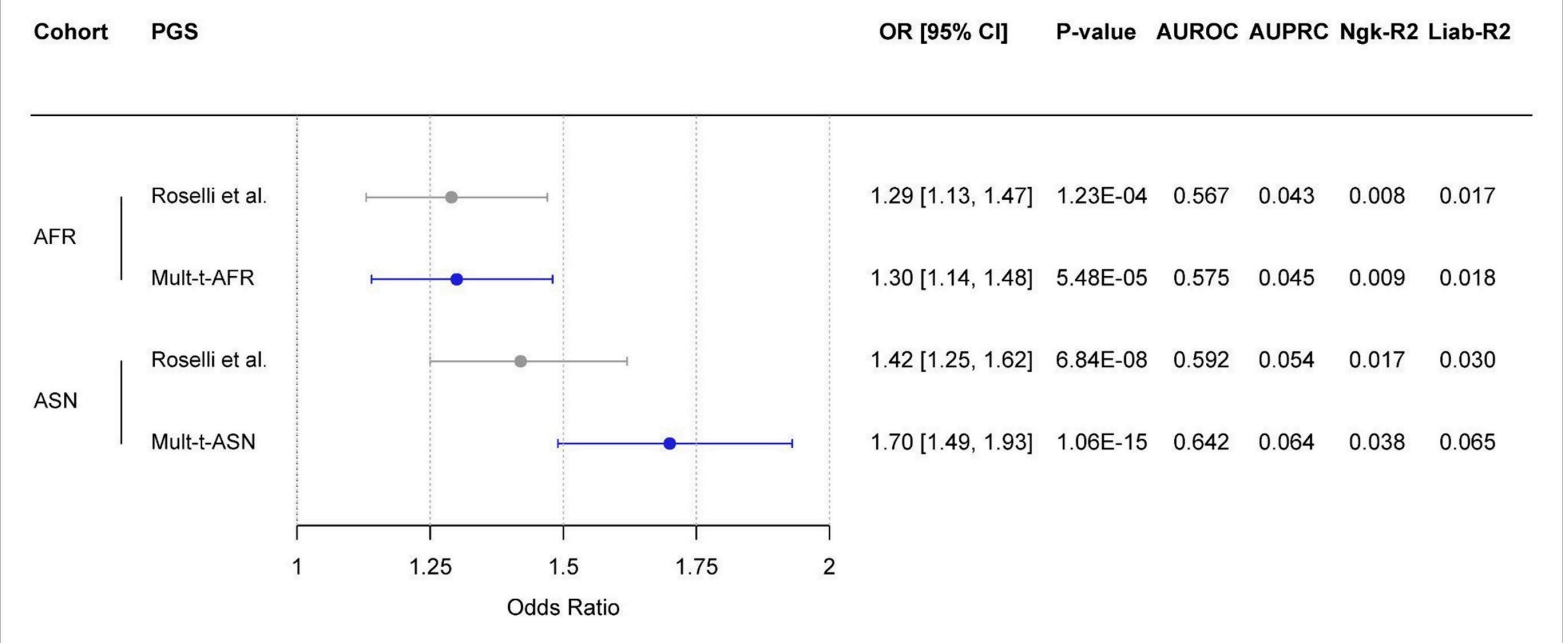
Comparison of the Roselli et al. and simplified non-European Mult-t polygenic scores within the UK Biobank. The simplified Mult-t scores assessed here excluded the traits height, BMI, and DCM, as these were based on either non-European UKB training data or exclusively European training data. The left panel displays a forest plot of the OR/SD increase in PGS for AF, with 95% CIs on the x-axis. The y-axis lists the ancestry-specific validation cohorts, ordered by decreasing cohort size, each paired with its respective PGSs. The right panel summarizes key performance metrics from the validation set. All estimates are based on logistic regression models adjusted for the first 20 PCs, age, and sex, using case/control counts from the validation dataset of the UK Biobank (Table S9). Model performance details are provided (Table S9).

We then evaluated the performance of a simplified version of the multi-trait PGSs, excluding traits built from non-European UK Biobank GWAS, among individuals of African and Asian ancestry from the UK Biobank. In both ancestry groups, the simplified Mult-t score outperformed the Roselli et al. score, with the most pronounced improvement observed in ASN ancestry (ΔOR/SD = 0.28; ΔAUROC = 0.050; ΔAUPRC = 0.010). In AFR, performance was modestly improved, primarily reflected by an enhanced AUROC score (ΔOR/SD = 0.01; ΔAUROC = 0.008; ΔAUPRC = 0.002) compared with the Roselli et al. PGS (Fig. 4). Additionally, the Mult-t scores showed higher explanatory power in both ASN and AFR populations, as reflected by increases in both the Nagelkerke R^2^, from 0.008 to 0.009 in AFR and from 0.017 to 0.038 in ASN, and the liability-scale R^2^, from 0.017 to 0.018 in AFR and from 0.030 to 0.065 in ASN (Fig. 4; Table S9). These data replicate the strong prediction improvement of our multi-trait PGS among ASN ancestry. For AFR, the relatively small improvement may be explained by the absence of BMI and height PGS in the simplified Mult-t PGS (Fig. 3).

### Performance comparison: The Mult-t-EUR vs Roselli et al. score

**Figure 5:**
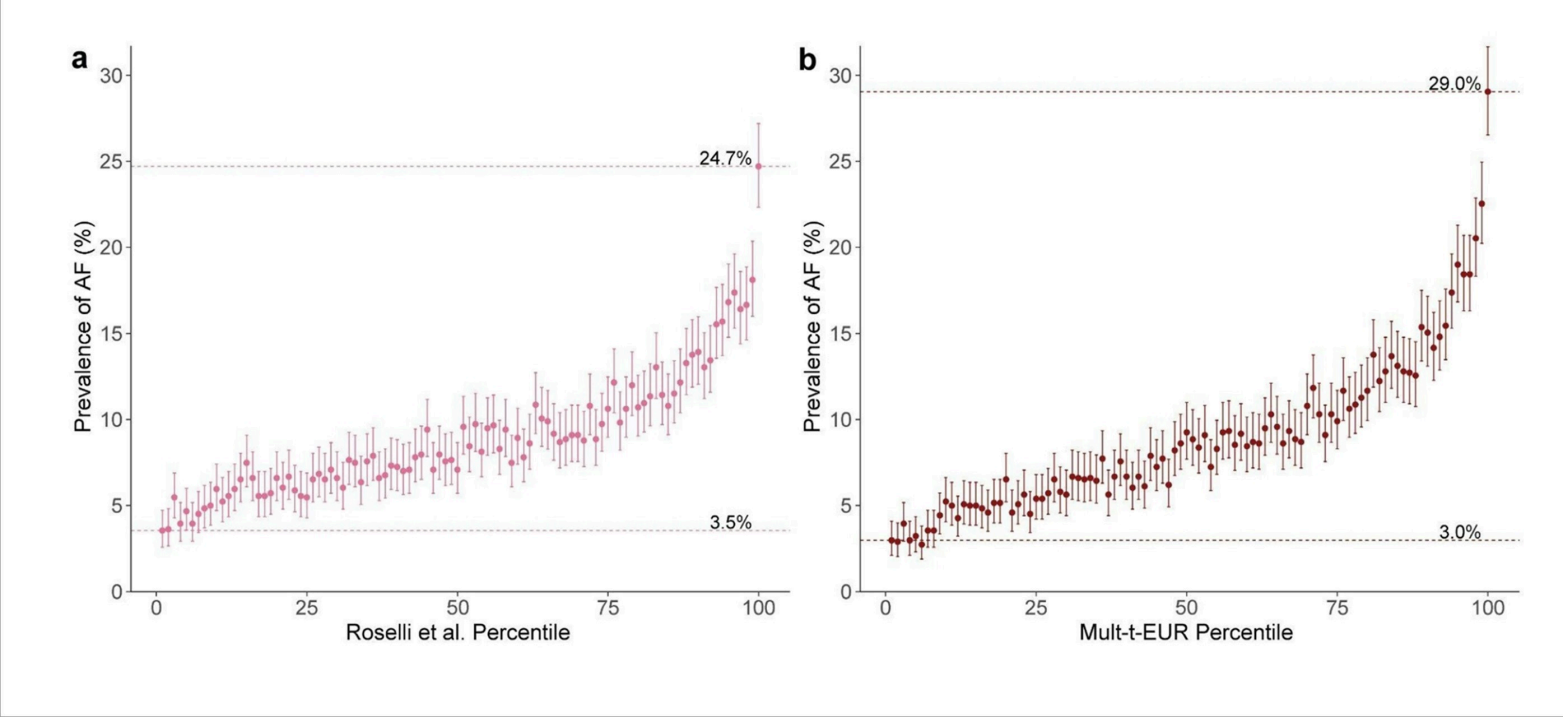
AF prevalence with 95% confidence intervals across 100 PGS percentile groups. Each panel shows the PGS percentile on the x-axis and AF prevalence with 95% CI on the y-axis. Dotted lines indicate AF prevalence in the lowest (bottom) and highest (top) percentiles. Participants were grouped by percentile using either the Roselli et al. PGS in pink **(a)** or the Mult-t-EUR PGS in dark red **(b)**. Both scores were evaluated in the 70% European ancestry validation dataset of All of Us, comprising 11,087 cases and 113,280 controls.

Finally, to further benchmark the performance of our new PGSs, we performed additional analyses to directly compare the Mult-t-EUR PGS and the Roselli PGS, within the European validation set of All of Us Research Program. Initially, we plotted AF prevalence with 95% confidence intervals (CIs) across 100 percentile-based strata, where participants were grouped by their percentile on either the EUR-tuned Mult-t or the Roselli PGS (Fig. 5). For the Mult-t-EUR score, prevalence ranged from 3.0% in the lowest percentile to 29.0% in the highest, compared to 3.5% and 24.7% for the Roselli score. These findings suggest that the multi-trait score more effectively concentrates AF cases in the highest risk group and reduces prevalence in the lowest, thereby improving overall risk stratification.

We also compared the distribution of PGS percentiles between AF cases and controls using both scores (Fig. S12). Among AF cases, the median percentile was higher for the Mult-t-EUR (69; interquartile range [IQR]: 43–88) than for the Roselli score (64; IQR: 38–86), while controls showed similar distributions, indicating stronger case stratification by the multi-trait model.

### Risk stratification at the extremes of the polygenic score distribution

To evaluate risk stratification at the extremes of the PGS distribution, we also compared individuals in the tails to those in the middle quintile of the population, using logistic regression. While the Mult-t PGS demonstrated improved stratification across the continuous range, differences between scores became particularly pronounced at the distribution extremes.

**Figure 6:**
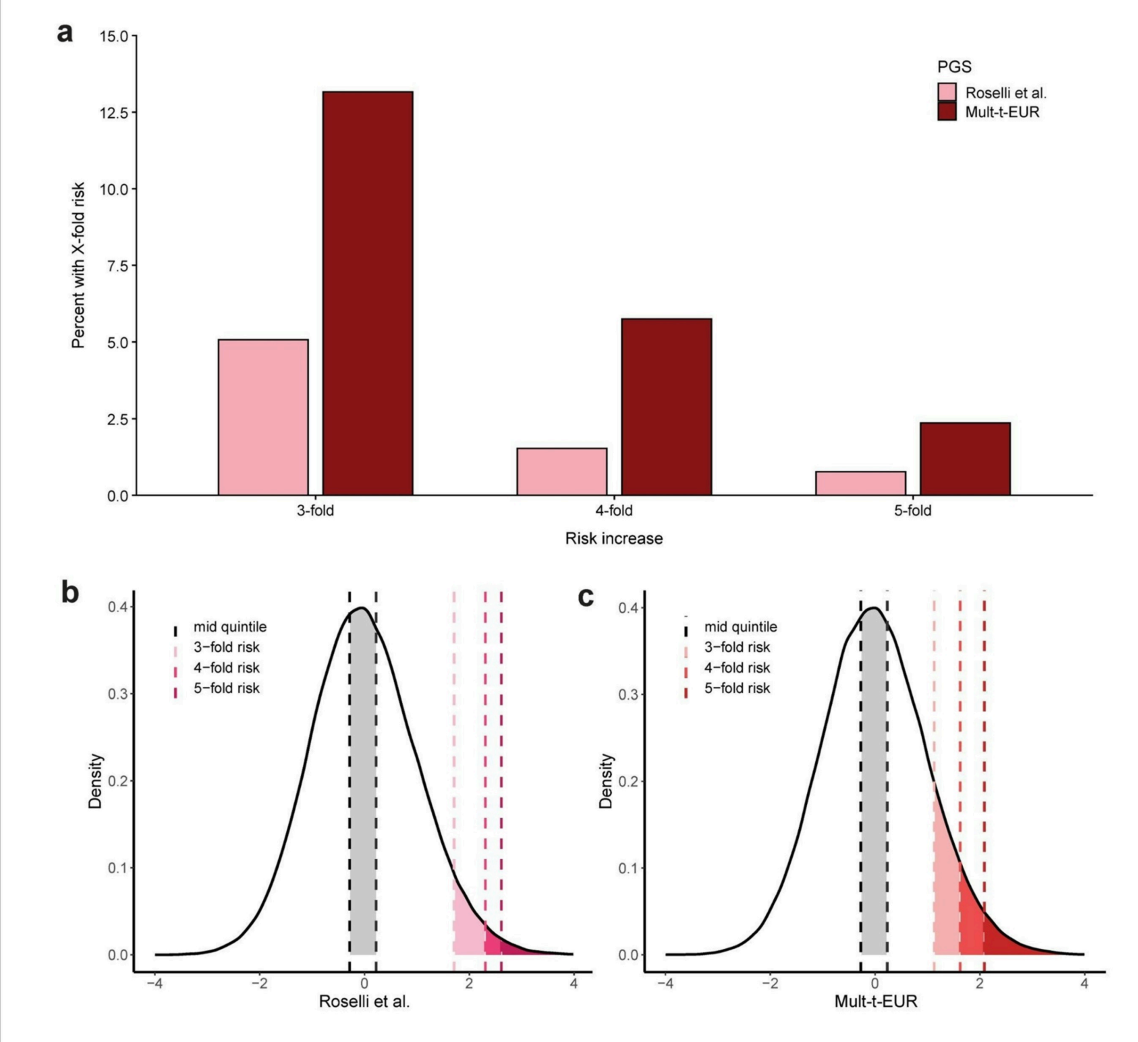
Proportion of individuals with 3-, 4-, or 5-fold increased AF risk compared to the middle quintile. All panels show the percentage of European ancestry participants in the 70% validation dataset of All of Us (11,087 cases / 113,280 controls) with a 3-, 4-, or 5-fold higher risk of AF relative to the middle PGS quintile. **a,** Bar plots display the proportions in each risk group for the Roselli et al. PGS (pink) and the Mult-t-EUR PGS (dark red). **b,c,** Density plots of the Roselli et al. (**b**) and Mult-t-EUR **(c)** PGSs. The x-axis displays polygenic score values, and the y-axis indicates density, representing the relative frequency of individuals with a specific polygenic score value. The middle quintile is shaded in grey, while regions corresponding to 3-, 4-, and 5-fold risk are highlighted in progressively darker tones. Odds ratios were estimated using logistic regression adjusted for age, sex, and the first 20 ancestry PCs.

To quantify this, we iteratively fitted the logistic regression models, progressively narrowing the upper tail to identify the smallest subgroups with at least a 3-, 4-, or 5-fold increased odds of AF compared to the middle quintile. Using this method, the Mult-t PGS captured a larger segment of the population at elevated risk than the Roselli score (Fig. 6a). Specifically, 13.2%, 5.8%, and 2.4% of individuals had 3-, 4-, and 5-fold increased odds of AF, respectively, with the Mult-t PGS, versus only 5.1%, 1.5%, and 0.8% with the Roselli score. Density plots further illustrate that high-risk individuals occupy a broader portion of the Mult-t distribution than in the Roselli distribution (Fig. 6b,c).

We applied a similar approach to identify individuals with substantially reduced risk. Focusing on a ⅓-fold decreased odds threshold, we iteratively expanded the lower tail until identifying the largest group meeting this criterion. More extreme thresholds, ¼- or ⅕-fold reduction, yielded group sizes too small for reliable interpretation. Interestingly, 7.0% of individuals had ⅓-fold reduced odds of AF with the Mult-t PGS, compared to only 0.6% with the Roselli score (Fig. S13a). Density plots visualize these low-risk segments (Fig. S13b,c).

These results indicate enhanced risk stratification at both ends of the distribution, as the Mult-t score more effectively shifts high-risk individuals toward the upper tail and low-risk individuals toward the lower tail, while reducing their presence in the middle quintile.

### Sensitivity analyses

We conducted an important sensitivity analysis for the assessment of the Roselli, ALLmeta, and Mult-t scores. In particular, we re-computed the performance metrics after excluding All of Us participants from Massachusetts (MA) - to avoid overlap with the AFGen cohort given the inclusion of Mass General Brigham cohorts in the base GWAS - and after excluding individuals with Veteran Affairs (VA) health coverage - to avoid overlap with the Million Veteran Program. In total, 3,048 AF cases and 24,670 controls were excluded. The resulting performance metrics (Fig. S14; Table S10) barely differed from the original results, indicating that the results were not driven by overlapping samples between the training and testing datasets.

Additionally, to assess variation in Mult-t score performance by subgroups, we performed a subset analysis of the Mult-t-EUR score stratified by sex and by age groups (45–54, 55–64, and 65–75 years) (Fig. S15; Table S11). Males exhibited a higher OR per SD compared to females (1.93 vs. 1.82), and the OR per SD increased progressively with age range (1.66, 1.76, and 1.95, respectively), indicating stronger genetic risk in males and with advancing age.

## Discussion

In this study, we aimed to construct improved polygenic scores for AF by combining several large multi-ancestry GWAS datasets with multiple novel methodological approaches. First, we used the SBaysesRC algorithm to create dense genome-wide PGSs from GWAS data from the AFGen Consortium and MVP. We found that the SBayesRC score, trained on a pan-ancestry meta-analysis, led to improved prediction as compared to the previous gold standard PGSs, with benefits seen across various ancestry groups. We subsequently evaluated two complementary strategies for multi-PGS construction: A multi-ancestry approach and a multi-trait approach. The multi-trait approach in particular provided improved prediction across diverse populations, especially among Asian ancestry, although prediction was still diminished when compared to European ancestry individuals. These findings allow for several conclusions.

First, we developed one of the strongest PGS for AF prediction in Europeans to date. The Mult-t-EUR PGS demonstrated a prediction with an OR per SD of 1.89 and a univariate AUROC of 0.646, among individuals of European ancestry. Notably, the Mult-t-EUR score showed improved case stratification in a direct comparison to both the PRSmix + PGS^2^ and the Roselli et al. PGS^12^, the latter shown to strongly improve prediction over the widely-used^20–22^ genome-wide PGS from Khera et al^23^. (**Supplementary Note**). Our score provided improved prediction both across the continuous score distribution and when comparing extremes to the middle quintile. Specifically, we identified 13.2%, 5.8%, and 2.4% of individuals in the extremes with 3-, 4-, and 5-fold increased risk of AF, respectively. These effect sizes are comparable to some of the strongest known clinical predictors of AF, such as heart failure (OR = 3.6)^24^, as well as to high-impact rare genetic variants in *TTN* (OR/HR = 2.06–4.41)^25–27^, which have substantially lower prevalences (~ 5% in middle-aged individuals^28^ and 0.44% in the general population^29^, respectively). Moreover, the Mult-t-EUR score explained 11% of the phenotypic variance in AF on the logit-liability scale, as compared to 5.9% explained by the Roselli et al. score. Nevertheless, we note that the Mult-t-EUR score still only captures half of the estimated common variant heritability of AF (estimated at approximately 22%^11^). These findings therefore illustrate how improved GWAS data and PGS methodology continue to improve the genetic prediction of AF, even in European ancestry, with additional improvements likely still possible in the future.

Second, our PGS framework provides important improvements to the genetic prediction of AF in non-European ancestries. Notably, our final Mult-t scores outperformed the Roselli et al. score across ancestry groups, reaching prediction with ORs per SD (95% CI) of 1.39 (1.32–1.45) in African ancestry, 1.45 (1.38–1.53) in Admixed-American ancestry, and 1.76 (1.56–1.99) in Asian ancestry. Our PGSs also surpassed recent multi-ancestry scores for AF developed by Gunn et al.^30^ (**Supplementary Note**). Consistent with data from Gunn et al., and consistent with data from other heritable traits^30,31^, we observed that all-ancestry training data improved AF prediction across ancestries. Nevertheless, prediction remains relatively diminished in African and Admixed-American ancestries, when compared to European ancestry. This finding may partially be explained by the relatively small sample size of non-European GWAS datasets for AF at this time, as well as poor transferability of European GWAS data to these ancestry groups^32–34^. In this light, we note that most AF PGSs currently available in the PGS Catalog remain derived from European ancestry-only cohorts^35–38^. Therefore, our novel ancestry-optimized PGSs represent an important step towards more equitable genomic prediction of AF, especially for Asian ancestry, although our findings also highlight the ongoing need for additional diverse GWAS cohorts.

Third, our work shows how PGS performance can be enhanced by integrating correlated traits within a multi-PGS framework. Specifically, we used a custom adaptation of the SBayesRC-multi tool (**Code Availability Statement**), which allowed for integration and ancestry-specific tuning of PGSs for multiple AF-related traits. This multi-trait approach yielded the largest relative gains among Asian (ΔOR/SD = 0.13; ΔAUROC = 0.004) and African (0.07; 0.007) ancestries, followed by European (0.07; 0.004) and Admixed-American (0.06; 0.004) ancestries. These findings suggest a particular utility of multi-trait PGSs, paired with ancestry-specific tuning, in underrepresented populations (**Supplementary Note**). We note that multi-trait approaches for PGSs have been explored in previous literature. For instance, one study applied LASSO to combine 937 European-trained PGSs, which yielded improved genomic prediction for several psychiatric disorders^39^. A landmark study on coronary artery disease integrated ancestry-specific PGSs and genetically-correlated traits - using stepwise model selection (stepAIC) within a European tuning set^19^ - leading also to substantially improved prediction. For AF, prior multi-trait approaches have used elastic net regression, including RFDiseasemetaPRS (OR/SD = 1.30)^40^ and PRSmix + ^2^ (liability-scale R^2^ = 0.07 among EUR, in our analyses). However, both of these models were evaluated exclusively in European-ancestry individuals in prior work. In contrast, our study shows the value of ancestry-informed multi-trait approaches for genomic prediction of AF across diverse populations.

We note that the PRSmix + PGS represents a score similar to the PGS that will be implemented clinically at MGB, as of late 2025^5,6^. Importantly, our Mult-t PGS substantially outperformed the currently-available PRSmix + PGS for AF (PGScatalog: PGS004706). Nevertheless, given that PRSmix + inherently leverages prediction power from other PGSs^2^, we acknowledge that future ancestry-specific optimization of PRSmix+ - incorporating also our PGSs - should prove fruitful to improve genomic prediction of AF further.

Fourth, our analyses highlight the power of the initial SBayesRC model^18^, a genome-wide Bayesian approach that incorporates functional annotations and leverages dense genome-wide markers. In previous work, SBayesRC showed superior prediction, compared to several other methods, across many heritable traits^41^. While genome-wide PGSs have previously been developed for AF^23,42^, most recent AF scores in the PGS Catalog remain limited, either not genome-wide or based on only ~ 1M SNVs^35,36,43^. In contrast, leveraging ~ 7M functionally-annotated variants, as we did, has been shown to substantially improve predictive accuracy as compared to using 1M unannotated SNVs^18^, further supported by substantially improved prediction using the SBayesRC model for AF in our analyses. Several other technical aspects of our PGS methodology are further discussed in the **Supplementary Note**.

We note that our study has several limitations. First, we were unable to create and validate separate Mult-t scores for East Asian and South Asian ancestries, given that no sufficiently-powered AF GWAS datasets for South Asians were available, and given that the East Asian validation sample in All of Us was too small. As such, further work is required to assess the predictive capacity of our PGS across different Asian sub-populations. Second, despite using genetically-predicted ancestry labels, our analyses are based on a cohort from the United States (and a cohort from the United Kingdom), meaning that some degree of European admixture is possible for the non-European individuals^44,45^. Additional work in continental cohorts is therefore required to replicate our findings, in the future. Third, due to the large contribution of European ancestry to the base GWAS data, the SBayesRC algorithm led to the largest prediction improvement among Europeans, despite our aim to reduce disparities across ancestries. We observed a specific order in our Mult-t score’s predictive performance (EUR > ASN > AMR > AFR), a pattern that closely mirrors the ancestry sample sizes reported in the base GWAS data and other large genetic datasets^46,47^. An exception was seen for African and Admixed-American ancestries, where African ancestry had larger GWAS sample size but lower PGS accuracy, a finding potentially due to greater genetic diversity and shorter haplotype blocks seen in Africans^48^. The reduced performance in these ancestries is not unique to our study, as similar trends have been reported in two CAD PGS studies^19,31^.

Looking ahead, we again stress that more adequately powered and diverse GWASs are needed, guided by key strategies outlined in a prior study^47^. This is particularly critical for AFR, AMR and SAS ancestries, which could be better represented through expanding efforts such as MVP^49^, H3Africa^50^, the Mexican Biobank^51^ and the Genes & Health initiative^52^, respectively, each of which require sustained funding to grow. Additionally, novel statistical methods that enhance cross-ancestry portability, such as SBayesRC, along with diverse evaluation cohorts like All of Us^53^, are essential for completing the PGS development process. In a similar fashion, additional work should assess how sex-stratified and sex-optimized PGSs may improve individualized genomic prediction (**Supplementary Note**).

As genomic prediction of AF improves, and as AF PGSs enter the clinic, it has to be considered how PGSs may be used to concretely improve AF care or screening. In recent work, an AF PGS was shown to enhance risk prediction when added to clinical models such as HARMS2-AF (AUROC: 0.828 to 0.839) and CHARGE-AF (0.808 to 0.828) in population-based cohorts like UK Biobank^14^, and in a clinical cohort of cardiovascular disease patients, where combining an AF PGS with CHARGE-AF and NT-proBNP improved the C-index from 0.67 to 0.70^54^. In CAD, prospective studies in primary care have demonstrated that adding PGSs to clinical scores improves prediction and is generally well-accepted by both clinicians and patients^55,56^. For AF, a care pathway can be envisioned where individuals with a high PGS are prioritized for aggressive risk factor management, as well as earlier routine AF screening^57^. Still, further research in real-world settings is needed to assess the clinical utility, implementation, and ethical considerations of AF PGSs across diverse populations. Furthermore, the field will need to define clear clinical use-cases (e.g. specific patient populations and indications) where PGS implementation stands to change standard care^1^. In the shorter term, the predictive utility of our improved PGSs should be assessed alongside established clinical risk models, for individuals of European and other genetic ancestries.

In conclusion, we demonstrate that integrating diverse GWAS data and correlated traits in a multi-PGS framework substantially improves genomic prediction of AF. Our multi-trait approach was particularly effective for individuals of Asian genetic ancestry, with further gains expected in other non-European ancestries as diverse training datasets expand. As AF PGSs enter the clinic, our ancestry-optimized scores may lay the groundwork for more equitable and interpretable genomic prediction of AF.

## Methods

### Meta-analysis of AF GWAS summary statistics

To improve the genomic prediction of AF across diverse ancestry cohorts, we increased the training sample size by combining genome-wide association study (GWAS) summary statistics from the AFGen and MVP cohorts using METAL. This tool performed an inverse-variance weighted meta-analysis, accounting for differences in standard errors, sample sizes, and allele frequencies between studies. Details on METAL’s input parameters, the execution script, and file pre- and post-processing steps are provided in the Supplementary Materials.

The following provides an overview of the cohorts included in the meta-analysis. AFGen, published by Roselli et al., is the largest meta-analysis to date focused on AF. It includes over 180,000 cases and approximately 1.5 million controls from multiple cohorts, with representation from AFR, Admixed American (AMR), and EAS ancestries^12^. The MVP is a U.S.-based cohort study comprising more than 635,000 individuals and providing GWAS summary statistics for over 1,200 traits, including AF. MVP also includes participants from AFR, AMR, and EAS backgrounds, enabling analyses across diverse ancestries^16^. Details on the case and control counts across ancestries are provided (Table S12).

### SBayesRC

To maximize the value of the diverse training data, we applied the SBayesRC algorithm (https://github.com/zhilizheng/SBayesRC)^58^, a Bayesian polygenic score tool that integrates functional genomic annotations to enhance prediction across ancestries. Specifically, SBayesRC (v0.2.6) incorporates data on 8,140,664 SNVs annotated with features such as enhancer and promoter status from the Baseline-LD v2.2^59^ model. For linkage disequilibrium (LD) reference, we used panels matched to the ancestry of the training data. Three LD panels were available, constructed from the imputed genotypes of UK Biobank participants of European (EUR; n = 347,800), African (AFR; n = 7,006), or East Asian (EAS; n = 2,252) ancestry. When no ancestry-matched LD panel was available, the EUR reference was used by default. An overview of SBayesRC’s input parameters, the execution script, and file pre- and post-processing steps is provided in the Supplementary Materials.

### Multi-ancestry polygenic score development

We first aimed to construct multi-ancestry PGSs (Fig. S1). To achieve this, we applied SBayesRC to ancestry-specific GWAS summary statistics (EUR, AFR, AMR, EAS) from the AFGen and MVP meta-analysis, or from AFGen alone for EAS. This generated ancestry-matched scores, denoted as EURmeta, AFRmeta, AMRmeta, and EASmeta. Subsequently, we used the SBayesRC-multi tool to combine each ancestry-specific score (e.g., EURmeta) with the all-ancestry score (ALLmeta). Specifically, we weighted both scores based on AF predictive performance in an ancestry-specific tuning set (Fig. S1). A detailed description on the SBayesRC-multi method, including the pipeline script, is provided in the Supplementary Materials. This approach was motivated by the hypothesis that combining the broad statistical power of the ALLmeta score with the ancestry-specific signal of, for example, the EURmeta score, while tuning within the corresponding ancestry group, would optimize predictive performance. Consequently, this approach yielded the multi-ancestry - or “Mult-a” - scores.

Due to the limited sample size in the 70% Asian validation set, we did not evaluate the EASmeta and Mult-a-ASN scores. Remarkably, as described in the first paragraph of the Results section, the other Mult-a scores closely matched the predictive performance of the simpler ALLmeta score in the EUR, AMR, or AFR validation subsets (Fig. S1). Therefore, we continued model development using the ALLmeta data.

### Multi-trait polygenic score development

To enhance predictive performance, we adapted the SBayesRC-multi tool to allow more flexible input and output handling, including the capacity to handle more than two PGS inputs (Fig. S3)^60^. A description of the differences between the original and adapted version of the tool is provided in the Supplementary Materials. We applied the adapted tool to combine the ALLmeta score with the all-ancestry trained PGSs from seven traits correlated with AF (Fig. S4). The traits were selected based on well described AF risk factors^61^, high genetic correlation in the Cardiovascular Disease Knowledge Portal^62^, and availability of large-scale GWAS data. The case/control counts for the training data on AF and correlated traits (HF, BMI, height, CAD, SBP, DCM, PR-interval) are listed in Table S12.

Unlike the multi-ancestry scores, we aimed to integrate information across traits rather than ancestries, making it unclear whether ancestry-specific or all-ancestry tuning would yield better performance. Therefore, we explored both approaches, generating multi-trait - or “Mult-t” - scores from ancestry-specific tuning and from all-ancestry tuning. Strikingly, as described in the second paragraph of the Results section, the ancestry-tuned Mult-t scores outperformed their all-tuned counterparts across the EUR, AMR, and AFR validation cohorts of All of Us (Fig. S4). Accordingly, the Mult-t-EUR, Mult-t-AFR, and Mult-t-AMR scores were selected as final models.

For the Mult-t-ASN score, we used an all-ancestry tuning set that excluded ASN individuals. This allowed us to retain a fully independent 100% ASN validation sample, which provided sufficient power for evaluation. However, because this non-ASN tuning approach would be counterintuitive for a multi-ancestry PGS, we did not apply it to generate the Mult-a-ASN score.

### Tuning and evaluation in All of Us and the UK Biobank

We used the All of Us dataset to tune the Mult-a and Mult-t models and to evaluate their predictive performance. Additionally, we assessed the previously published PGS by Roselli et al., obtained from the Cardiovascular Disease Knowledge Portal, as well as the PRSmix + PGS for AF (PGScatalog: PGS004706). The All of Us Research Program is a U.S.-based initiative aimed at improving health care for everyone by building a large-scale and ancestrally diverse biomedical database. For this study, we used version 8 of the dataset, including only individuals with both high-quality short-read whole genome sequencing (srWGS) and electronic health record (EHR) data, representing 76.8% of the 410,401 srWGS participants. Participants were randomly split into a 30% tuning set and a 70% testing set and labeled according to their genetically inferred ancestry: European, African, Admixed American, or Asian (the latter combining East and South Asian groups due to small sample sizes). Ancestry-specific case/control numbers for both sets are provided in Table S4. AF cases were broadly defined to include both atrial fibrillation and atrial flutter, with exact definitions specified by OMOP IDs listed in Table S13. Details on the All of Us variant- and sample-level quality control procedures and ancestry inference are provided in the **Supplementary Note**.

We also evaluated the Roselli et al. PGS and simplified versions of the non-European final Mult-t models within genetically inferred AFR and ASN ancestry validation sets of the UK Biobank (UKB). For the simplified models, trait GWAS that included non-European UKB data were excluded to avoid overlap between training and testing sets. Details on these scores, as well as variant- and sample-level quality control, ancestry inference, AF phenotype definitions, and PGS evaluation within the UKB, are described in the **Supplementary Note**.

Polygenic scores were computed using the ‘--score’ function in PLINK2. To account for population structure, each PGS was regressed on the first 20 principal components (PCs) within the all-ancestry testing set, and the residuals were extracted to remove PC effects. The residualized PGSs were then standardized to mean zero and SD one, after which ancestry-specific subsets were extracted. Finally, the PGS’s ability to distinguish AF cases from controls was assessed using logistic regression, and several performance metrics were derived. The (log-)odds ratio, P-value and R^2^ were derived from multivariate logistic regression models corrected for age and sex; AUC and AUPRC values were derived from univariate logistic regression models. For R^2^, we used Nagelkerke’s and liability R^2^, calculated as delta R^2^ (ΔR^2^ = R^2^_full − R^2^_base) divided by the residual R^2^ (1 − R^2^_base). Liability R^2^ was estimated using the ancestry-specific AF prevalence within the AoU dataset^63^.

## Supplementary Material

Supplementary Files

This is a list of supplementary files associated with this preprint. Click to download.

• ImprovedAFPGSacrossdiverseancestriessupplementarytables.xlsx

• ImprovedAFPGSacrossdiverseancestriessupplementarymaterials.docx

## Data Availability

We downloaded the all-ancestry Roselli et al. PGS from the Cardiovascular Disease Knowledge Portal under the weblink https://cvd.hugeamp.org/downloads.html#polygenic. The PRSmix+ PGS for AF was obtained from the PGS catalog (PGS004706). The four ancestry-tuned Mult-t PGSs are available in the PGS Catalog under accession ID **[insert ID]**, as is the ALLmeta PGS **[insert ID]**. Additionally, the non-EUR PGSs—excluding traits trained on UK Biobank data—can also be accessed in the PGS Catalog under accession ID **[insert ID]**. Detailed information on the GWAS training datasets, including article references and links to the data sources, is provided in Table S12. Pan-ancestry and ancestry-specific GWAS summary statistics from the AFGen and MVP meta-analysis will be made available upon publication, either through dbGAP or the Cardiovascular Disease Knowledge Portal.

## Software and code availability statement

Quality control was performed with various software tools as described in the Supplementary Materials, and mostly using PLINK2 (https://www.cog-genomics.org/plink/2.0/; various versions from May 2025 release onwards). Meta-analysis of GWAS was performed using METAL (release 2011–03-25) (https://csg.sph.umich.edu/abecasis/metal/download/). Execution scripts for the AF (https://github.com/poeyahay/AFib_PGS/blob/main/METAL/AFGen%2BMVP.sh) and SBP (https://github.com/poeyahay/AFib_PGS/blob/main/METAL/SBP_trait.sh) summary statistics are available. Polygenic scores were generated with SBayesRC (v0.2.6) (https://github.com/zhilizheng/SBayesRC) and PLINK2 (https://www.cog-genomics.org/plink/2.0/; various versions from February 2025 release onwards). Code for running SBayesRC on the all-ancestry AF summary statistics is provided (https://github.com/poeyahay/AFib_PGS/blob/main/SBayesRC/SBRC_Run.sh). For incorporation of multiple polygenic scores, we used the SBayesRC-multi tool (https://github.com/poeyahay/AFib_PGS/blob/main/Multi/SBRCmulti.R) and our adapted multi tool version (https://github.com/poeyahay/AFib_PGS/blob/main/Multi/Multi_Tool.R), both run in R v4.5.0 and available as open-source software on GitHub. Performance metrics were extracted from the logistic regression models primarily using base R. AUROC was calculated with the pROC package, AUPRC with the PRROC package, and Nagelkerke’s R^2^ with the fmsb package. Liability R^2^ was derived using a custom function based on formulas described in Lee et al. (2012). Analyses, data pre- and post- processing steps, and figure generation performed in R were run using versions 4.4.1–4.5.0.
